# Pulmonary outcomes in adults with a history of Bronchopulmonary Dysplasia differ from patients with asthma

**DOI:** 10.1186/s12931-019-1075-1

**Published:** 2019-05-24

**Authors:** Petra Um-Bergström, Jenny Hallberg, Melvin Pourbazargan, Eva Berggren-Broström, Giovanni Ferrara, Maria J. Eriksson, Sven Nyrén, Jing Gao, Gunnar Lilja, Anders Lindén, Åsa M. Wheelock, Erik Melén, C. Magnus Sköld

**Affiliations:** 1 Sachs’ Children and Youth Hospital, Department of Pediatrics, Södersjukhuset, 118 83 Stockholm, Sweden; 2 Department of Respiratory Medicine & Allergy, Karolinska University Hospital, Stockholm, Sweden; 3 Department of Medicine, Karolinska Institutet, Solna, Stockholm Sweden; 4 Institute of Environmental Medicine, Karolinska Institutet, Stockholm, Sweden; 5 Department of Clinical Science and Education, Södersjukhuset, Karolinska Institutet, Stockholm, Sweden; 6 Department of Clinical Physiology, Karolinska University Hospital, Stockholm, Sweden; 7 Department of Molecular Medicine and Surgery, Karolinska Institutet, Stockholm, Sweden; 8 Thoracic Radiology, Karolinska University Hospital, Stockholm, Sweden

**Keywords:** Bronchopulmonary dysplasia, Adults, Astma, Lung function tests, Oscillometry, Spirometry, Multiple breath washout, HRLQoL

## Abstract

**Background:**

Bronchopulmonary dysplasia (BPD) is a risk factor for respiratory disease in adulthood. Despite the differences in underlying pathology, patients with a history of BPD are often treated as asthmatics. We hypothesized that pulmonary outcomes and health-related quality of life (HRQoL) were different in adults born preterm with and without a history of BPD compared to asthmatics and healthy individuals.

**Methods:**

We evaluated 96 young adults from the LUNAPRE cohort (clinicaltrials.gov/ct2/show/NCT02923648), including 26 individuals born preterm with a history of BPD (BPD), 23 born preterm without BPD (preterm), 23 asthmatics and 24 healthy controls. Extensive lung function testing and HRQoL were assessed.

**Results:**

The BPD group had more severe airway obstruction compared to the preterm-, (FEV_1−_ 0.94 vs. 0.28 z-scores; *p* ≤ 0.001); asthmatic- (0.14 z-scores, *p* ≤ 0.01) and healthy groups (0.78 z-scores, *p* ≤ 0.001). Further, they had increased ventilation inhomogeneity compared to the preterm- (LCI 6.97 vs. 6.73, *p* ≤ 0.05), asthmatic- (6.75, *p* = 0.05) and healthy groups (6.50 p ≤ 0.001). Both preterm groups had lower D_LCO_ compared to healthy controls (*p* ≤ 0.001 for both). HRQoL showed less physical but more psychological symptoms in the BPD group compared to asthmatics.

**Conclusions:**

Lung function impairment and HRQoL in adults with a history of BPD differed from that in asthmatics highlighting the need for objective assessment of lung health.

**Electronic supplementary material:**

The online version of this article (10.1186/s12931-019-1075-1) contains supplementary material, which is available to authorized users.

## Background

In adults, chronic obstructive lung disease (COPD) caused by cigarette smoke [1] represent the vast majority of these patients. However, it has been acknowledged that 20–25% of all COPD patients have never smoked [2, 3]. Other risk factors include asthma, recurrent respiratory infections, air pollution, biomass smoke and occupational exposures as well as dietary habits [4]. In addition, early life events have been shown to be associated with development of chronic airway obstruction [5]. Thus, preterm birth [6], low birth weight and restricted growth pattern [7, 8], maternal factors [9] and genetic predisposition have all been discussed in this context [10, 11]. Infants born before gestational week 30 and with birth weight less than 1000 g are particularly vulnerable and approximately 10–30% of these children will develop bronchopulmonary dysplasia (BPD), a form of neonatal chronic lung disease [12]. Patients with BPD are often regarded as having asthma and are frequently treated with asthma medications without any scientific evidence for this assumption [13].

We hypothesized that adults born preterm, in particular those with a history of BPD, have a respiratory impairment that is different from what is observed in asthma, and that this significantly affects quality of life. To test this hypothesis, we examined in a cross-sectional setting never-smoking adults born preterm with and without a previous diagnosis of neonatal BPD and compared them to patients with asthma and healthy individuals. We thus aimed to characterize functional abnormalities in the airways and lung parenchyma and to correlate the findings to symptoms, quality of life and demographics.

## Methods

### Participants

We included four adult study groups: preterm born at gestational age (GA) ≤ 32 weeks with a neonatal diagnosis of BPD (BPD), preterm (born ≤32 weeks) without BPD (preterm), patients (born ≥37 weeks) with asthma according to GINA guidelines [14] (asthma) and healthy controls (born ≥37 weeks) as part of the LUNAPRE (**LU**Ng obstruction in **A**dulthood of **PRE**maturely born) study (clinicaltrials.gov/ct2/show/ NCT02923648).

The preterm born participants with and without a history of BPD were recruited from a pre-existing cohort [15] at the neonatal unit of Sachs’ Children and Youth Hospital, Södersjukhuset, Stockholm, Sweden, where they were admitted in the neonatal ward between 1992 and 1998. Tracing and recruitment to LUNAPRE are detailed in Additional file 1: Figure S1. All participants provided written informed consent, and the study was approved by the regional ethics committee in Stockholm (ref: 201211872–31/4).

Between 2013 and 2017, patients and controls were invited to Department of Medicine Solna, Karolinska Institutet, Department of respiratory medicine and allergy, Karolinska University Hospital Solna, Stockholm, Sweden, and at Sachs’ Children and Youth Hospital, Södersjukhuset, Stockholm.

The diagnosis of BPD was based on the need for supplemental oxygen for at least 28 days and severity degree determined at 36 weeks GA according to Jobe and Bancalari [16]. The diagnosis of allergic asthma [14] was confirmed by a positive methacholine challenge test with a decrease in FEV_1_ ≥ 20% and presence of IgE sensitization to any airborne allergen (see below). None of the participants used inhaled corticosteroids, leukotriene receptor antagonists or antihistamines within 3 months prior to inclusion. The asthma group was not examined during pollen season. Information on perinatal and neonatal history was collected from the Swedish Medical Birth Registry and medical charts. This included information on maternal smoking during pregnancy, multiple birth, caesarean section, treatment with prenatal steroids, Apgar score, GA at birth, birth weight (BW), instillation of surfactant, number of days on a ventilator, Continuous Positive Airway Pressure and supplemental oxygen, Retinopathy of Prematurity (ROP) and Patent Ductus Arteriosus (PDA). Small for gestational age (SGA) was defined according to Marŝàl as -2SD [17].

Venous blood samples were drawn for analysis of white blood cell differential counts, C-reactive protein, alpha-1 antitrypsin, and an in vitro screening for IgE-sensitization towards airborne allergens, Phadiatop® (Thermo Fisher Scientific; Pharmacia, Uppsala, Sweden). Phadiatop® includes analysis of IgE-antibodies against birch, timothy, mugwort, cat, dog, horse dander, mold (*Cladosporium herbarum*), and house dust mite (*Dermatophagoides pteronyssinus*). Analyses were done at the clinical laboratory of Karolinska University Hospital, Solna, Sweden.

### Health-related quality of life and symptoms

The validated Swedish version of the 36-item Short-Form Health Survey, first version (SF-36) [18] was used, a generic instrument for measuring health-related quality of life (HRQoL). Also, St George’s Respiratory Questionnaire (SGRQ) [19] was employed, a disease specific 50-item questionnaire designed to measure impact on overall health, daily life, and perceived well-being in patients with obstructive airways disease. All participants also answered questions including environmental exposures, education, lifestyle, tobacco use, former and present health conditions, symptoms, and pharmacological treatment.

### Lung function testing

Lung function testing was performed according to American Thoracic Society/European Respiratory Society guidelines [20–23]. Lung function testing included dynamic spirometry (Sensormedics 6200, SensorMedics, Yorba Linda, California, USA) where the highest values of forced expiratory volume in 1 s (FEV_1_) and forced vital capacity (FVC), were extracted and FEV_1_/FVC ratio calculated and used for analysis. Whole body plethysmograph and diffusing capacity (Vmax62 J CareFusion, SensorMedics) provided information on residual volume (RV), vital capacity (VC), total lung capacity (TLC) and diffusing capacity of the lung for Carbon monoxide (D_LCO_). Methacholine challenge test (Spira nebulizer, Spira Elektro 2, Respiratory Care Centre, Hämeenlinna, Finland) was used to decide hyper-reactive airways. The mean value of frequency dependence of resistance (R_5–20_) and the square root of the area of reactance (AX^0.5^) measured by impulse oscillometry (IOS) (Jaeger MasterScreen-IOS system, Carefusion Technologies) were used for analysis. Mean values for lung clearance index (LCI) were extracted by Nitrogen dioxide multiple breath washout (N_2_ MBW) (Exhalyzer®D N_2_ MBWdevice, Eco Medics AG, Duernten, Switzerland). Fractional exhaled nitric oxide *(*FeNO) was measured (EcoMedics Exhalyzer® CLD 88sp with Denox 88, Eco Medics, Duernten, Switzerland). In a few study subjects FeNO was measured using a NIOX device (Aerocrine AB, Solna, Sweden) [24]. Supplementary details on the lung function testing are provided in Additional file 2.

### Statistical analysis

Demographic data are presented as median and range for continuous variables, or numbers and percentages for categorical variables. Due to non-normally distributed data, comparisons between groups were performed using the Wilcoxon rank-sum test for continuous variables. The Pearson’s χ-squared test was used for categorical outcomes. FVC, FEV_1_ and FEV_1_/FVC were converted to z-scores using the Global Lung Initiative reference values (GLI) [25]. Cross-sectional comparisons between groups were made using the Wilcoxon rank-sum test. Associations between other lung function outcomes and the four groups were analyzed using linear regression on the median, adjusting for sex, height, and age when appropriate [26] . Correlations were assessed with Spearman’s test. Correction for multiple testing was performed with false discovery rate by Benjamini-Hochberg. *P*-values of < 0.05 were considered statistically significant. Analyses were performed with the Stata 13.1 software package (StataCorp LP, College Station, TX, USA).

## Results

### Participants

Twenty-nine individuals in the BPD group and 28 individuals in the preterm group accepted participation in the study. Three subjects in the BPD group and five in the preterm group were excluded due to tobacco smoking resulting in a final inclusion of 26 and 23 individuals respectively. Only two subjects in the BPD group used inhaled corticosteroids prior to inclusion and these were included after a wash-out period of 3 months. The asthma group included 23 and the healthy control group 24 subjects. Participant characteristics are summarized in Table 1. There was a significant (*p* < 0.001) lower proportion of individuals sensitized for IgE in the BPD group comparing to the preterm group. The asthma group had significantly (*p* < 0.001) higher blood eosinophil counts than did the other groups (Table 1).

**Table 1:** Characteristics of study participants

	BPD *n* = 26	Preterm *n* = 23	Asthma *n* = 23	Healthy controls *n* = 24
Sex, Male/Female	11/15	10/13	10/13	12/12
Age, years	19.6* (18.2;21.2)	19.1** (18.3;22.4)	20.2 (18.6;23.3)	20.5 (18.3;23.8)
Weight, kg M/F	69.6/61.5 (48;109.9)/(44.5;88)	68/59.2 (55;85.5)/(44;72.4)	75.7/59 (62;89)/(48;106)	71.8/ 62 (56.5;117.4)/(44.8;112)
Length, cm M/F	175/166 (155;192)/(155;173)	182/165 (169;192)/ (152;173)	174/168 (164;182)/(150;176)	180/167 (168;194)/(151;190)
BMI, m/kg^2^ M/F	20/22 (18;30)/(18;32)	20/ 21 (17;28)/(17;28)	24/21 (21;29)/(18;34)	21.5/22 (20;33)/(18;38)
FeNO, ppb	14.9 (9.2;22.2)	13.1 (11;19.8)	31.4** (11.6;49.2)	10.9 (9.7;17.1)
Positive Phadiatop	3 (12)	8 (35)	23 (100)	0
Blood Eosinophils 1 × 10^2^/μL	0.1 (0.0;0.3)	0.0 (0.0;0.2)	0.3*** (0.0;0.7)	0.0 (0.0;0.5)
CRP, mg/L	0.35 (0;2.1)	0.32 (0;1.3)	0.46 (0;7.6)	0.34 (0;7.7)
Alpha-1 antitrypsin g/L	1.4 (0.8;1.7)	1.3 (1.0;1.7)	1.4 (1.1;1.8)	1.4 (1.1;1.9)
Antidepressant medication	1 (3.8)	2 (8.7)	2 (8.7)	1 (4.1)
ADHD/ADD medication	3 (11.5)	3 (13)	0	1 (4.1)

Data are presented as median (range) or numbers (%). Abbreviations: *BPD* bronchopulmonary dysplasia, *M* male, *F* female, *BMI* body mass index, *FeNO* fractional exhaled nitric oxide, *CRP* C-reactive protein, *ADHD* Attention deficit hyperactivity disorder, *ADD* Attention deficit disorder

*p ≤ 0.05, **p ≤ 0.01, ***p ≤ 0.001 (compared to healthy)

Perinatal characteristics are summarized in Table 2. Subjects in the BPD group had lower GA and BW compared to preterm group (*p* < 0.001). The number of subjects born SGA was according to references in both BPD- and preterm groups [17]. Delivery by Caesarean section was more common in the groups of preterm born individuals, 78 and 50%, respectively, in the preterm- and BPD groups (*p* = 0.043 compared to healthy controls). There was a significantly higher proportion of individuals treated for PDA and having septicemia during the neonatal period in the BPD group compared to the preterm group (Table 2). The included subjects in the BPD- and preterm groups did not differ in BW, GA and SGA from the corresponding groups in the whole original cohort [15] except the BPD group who had 4 days lower mean GA.

**Table 2:** Perinatal characteristics of study participants

	BPD *n* = 26	Preterm *n* = 23	Asthma *n* = 23	Healthy controls *n* = 24
Male/Female	11/15	10/13	10/13	12/12
Gestational age at birth, weeks	26^###^ (24–31)	29.5 (26–32)	40 (38–42)	40 (37–43)
Birth weight, g	960^###^ (583–1510)	1470 (659–2200)	3505 (2660–4840)	3458 (2670–4550)
Small for gestational age	8 (31)	7 (30)	0	0
Maternal smoking during pregnancy	7 (27)	3 (13)	2 (9)	5 (21)
Prenatal corticosteroid therapy	11 (42)	10 (43)	N/A	N/A
Caesarean section	13 (50)	18 (78)	1 (4)	1 (4)
Multiple birth	8 (31)	12 (52)	0	0
Apgar score at 1 min	5 (1–9)***	8 (4–10)	9 (5–10)	9 (5–9)
Apgar score at 5 min	7 (3–10)***	9 (3–10)**	10 (7–10)	10 (8–10)
Instillation of surfactant	8 (31)	2 (4)	N/A	N/A
Ventilator therapy, days	4 (0–38)	0 (0–8)	N/A	N/A
CPAP, days	41 (3–70)	3 (0–19)	N/A	N/A
Supplemental O_2_, days	67 (28–180)	3 (1–26)	N/A	N/A
Septicemia	16 (62)^##^	6 (26)	N/A	N/A
PDA, treated	8 (31)^##^	0	N/A	N/A
ROP grade 3–4	7 (27)	3 (13)	N/A	N/A

Data are presented as median (range) or numbers (%). Abbreviations: *BPD* bronchopulmonary dysplasia, *N/A* not applicable, *CPAP* continuous positive airway pressure, *PDA* patent ductus arteriosus, *ROP* retinopathy of prematurity

^##^*p* ≤ 0.01; ^###^*p* ≤ 0.001 (BPD versus preterm)

**p ≤ 0.01; ***p ≤ 0.001 (Comparing BPD-, preterm- and asthma-groups to healthy controls)

### Health-related quality of life and symptoms

**Fig. 1:**
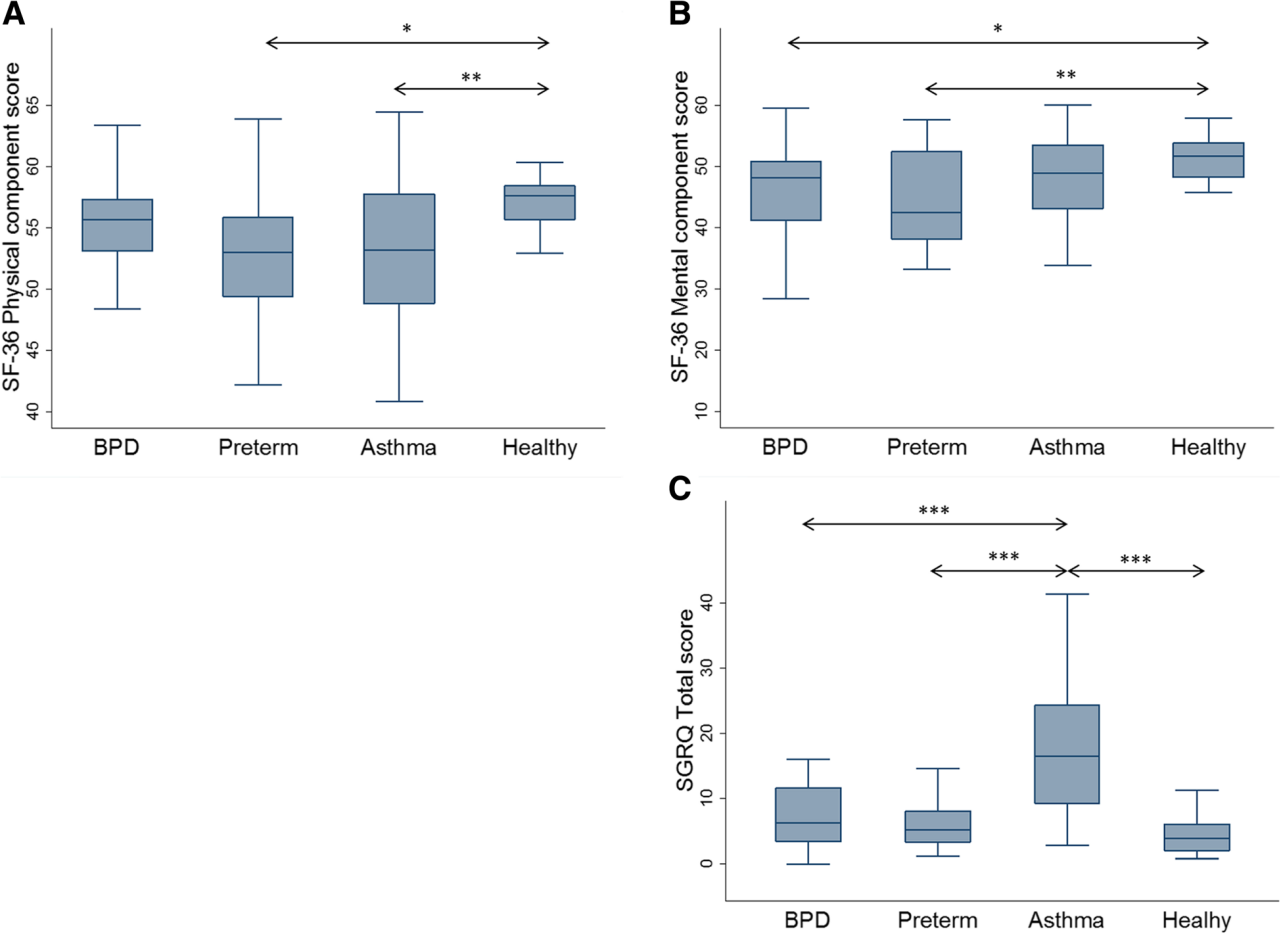
Graphical summery of health related quality of life questionnaires. The boxplots show the median values and IQR for (**a**): SF-36 physical component, (**b**): SF-36 mental component and (**c**): SGRQ total score. *: *p* ≤ 0.05; **: *p* ≤ 0.01; ***: *p* ≤ 0.001 Abbreviations: BPD: bronchopulmonary dysplasia; IQR: inter quartile range; SF-36: 36-item Short-Form Health Survey; SGRQ: Saint George’s Respiratory Questionnaire

The physical component summary (PCS) scores of SF-36 showed no significant difference between healthy controls and the BPD group, but both preterm- and asthma groups reported lower scores (*p* < 0.01 and *p* < 0.05) than the healthy controls. Both BPD- and preterm groups reported decreased mental component summery (MCS) scores (Fig. 1a and b; Additional file 3: Table S1).

The asthma group had significant higher total score in SGRQ (*p* < 0.001) compared to the healthy controls, BPD- and preterm groups. There were no significant differences between BPD-, preterm groups and healthy controls (Fig. 1c; Additional file 3: Table S1).

The asthma group reported more wheeze and cough than the other groups. It was more common in the BPD- and asthma groups to have phlegm that was difficult to expectorate compared to the healthy controls. There were no significant differences between the groups in frequency of pneumonia, hours of physical activities or sleeping hours. The asthma group spent less time in front of a screen (TV, computer) (Additional file 3: Table S1).

### Lung function

**Fig. 2:**
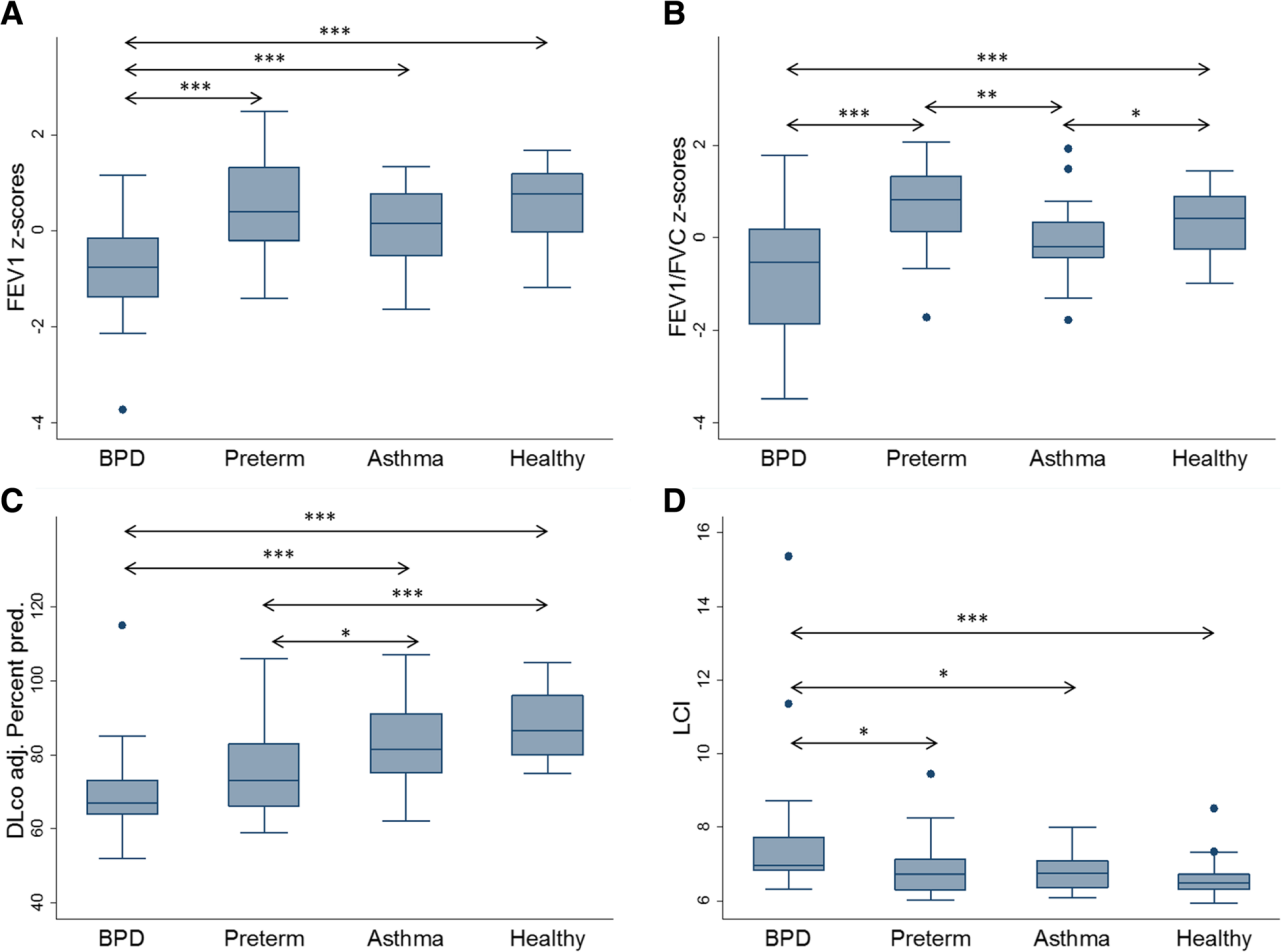
Graphical summery of airflow, ventilation inhomogeneity and gas diffusion. The boxplots show the median values and IQR for (**a**): FEV_1_; (**b**): FEV_1,_/FVC ratio, (**c**): D_Lco_ and (**d**): LCI .*: *p* ≤ 0.05; **: *p* ≤ 0.01; ***: *p* ≤ 0.001 Abbreviations: BPD: bronchopulmonary dysplasia; IQR: inter quartile range; FEV_1_: forced expiratory volume in 1 s; FVC: forced vital capacity; D_Lco adj_: diffusing capacity of the lung for carbon monoxide, adjusted for haemoglobin; LCI: lung clearance index

The BPD group had a decreased FEV_1,_ FVC and FEV_1_/FVC ratio compared to the other groups. The asthma group had decreased FEV_1,_ FVC and FEV_1_/FVC ratio compared to healthy controls and the preterm group (Table 3; Fig. 2a and b). Twenty percent of the BPD group were reversible to bronchodilator whereas 11% were reversible in the preterm- and 14% in asthma-groups. There were no statistically significant differences in reversibility between the BPD- and the asthma groups (Table 3). In the BPD group, 27% had a FEV_1_/FVC ratio below lower limit of normal (≤ − 1.64 z-scores). The corresponding percentage in the preterm- and in the asthma groups was 4.6 and 4.6% respectively (Table 4).

**Table 3:** Lung function in each study group

	BPD *n* = 26	Preterm *n* = 23	Asthma *n* = 23	Healthy controls *n* = 24
FEV_1_ *z-scores*^a^	−0.94*** (− 1.57; −0.08)	0.28 (−0.27; 1.18)	0.14 (−0.52; 0.77)	0.78 (−0.03; 1.26)
FEV_1_% pred.^a^	88.9*** (81.4; 99.1)	103.3 (96.9; 113.3)	101.7 (94.1; 109.0)	108.9 (99.7; 114.1)
FVC *z-scores*^a^	−0.32* (− 1.24; 0.14)	− 0.26 (− 0.64; 0.60)	0.13 (− 0.48; 0.85)	0.37 (− 0.18; 0.85)
FVC % pred.^a^	96* (85.6; 101.7)	96.9 (92.2; 107.5)	101.6 (94.5; 110.4)	104.6 (97.9; 109.8)
FEV_1_/FVC^b^	0.82*** (0.79; 0.85)	0.91 (0.87; 0.94)	0.86 (0.83; 0.89)	0.90 (0.87; 0.93)
FEV_1_/FVC *z-scores* ^a^	−0.67*** (−1.86; 0.18)	0.82 (0.13; 1.33)	−0.20* (− 0.43; 0.33)	0.42 (− 0.26; 0.89)
Reversibility %	6.9 (3.3; 10.9)	3.7 (1.4; 7.6)	5.9 (3.7; 8.3)	4.3 (1.9; 6.2)
Methacholine challenge pos.	19 (73)	13 (56)	23 (100)	0
TLC % pred.^a^	95* (87.5; 101.5)	97 (92; 114)	99 (95; 106)	104 (95.5; 108)
RV % pred.^a^	88 (60.5; 99.5)	79 (65; 132)	72.5 (52; 96)	84.5 (68.5; 105.5)
VC % pred.^a^	96.5** (88; 106)	103 (96; 110)	106 (99; 113)	107 (100.5; 115.5)
RV/TLC	20 (17; 24.5)	20.5 (17.5; 22.5)	18.5 (14; 23)	19 (17; 22.5)
D_LCO adj_ % pred.^a^	67*** (64; 73)	73*** (65; 83)	81.5 (75; 91)	86.5 (80; 96)
LCI^a^	6.97*** (6.84; 7.72)	6.73 (6.29; 7.12)	6.75 (6.37; 7.08)	6.50 (6.31; 6.72)
R_5–20_ (kPa/L/sec)^b^	0.014 (−0.002; 0.030)	0.021 (0.003; 0.039)	0.015 (−0.003; 0.033)	0.007 (− 0.010; 0.024)
AX^0.5^ (kPa/L)^b^	0.156 (0.120; 0.192)	0.163 (0.123; 0.202)	0.140 (0.099; 0.181)	0.125 (0.086; 0.164)

BPD-, Preterm- and asthma- groups were compared to healthy controls. Data are presented as median (IQR) numbers (%). All dynamic spirometry measures (FEV_1_, FVC, FEV_1_/FVC) and the impulse oscillometry data (R_5–20_, AX) are post bronchodilator (four doses of salbutamol 0.1 mg/dose) measures

^a^Analysis with quantile regression on median

^b^Analysis with quantile regression on median adjusting for age, sex and height

*p ≤ 0.05; **p ≤ 0.01; ***p ≤ 0.001, comparing BPD-, preterm- and asthma-groups to healthy controls

Abbreviations: *BPD* bronchopulmonary dysplasia, *FEV*_*1*_ forced expiratory volume in 1 s, *FVC* forced vital capacity, *TLC* total lung capacity, *RV* residual volume, *VC* vital capacity, *D*_*LCO adj*_ diffusing capacity of the lung for Carbon monoxide adjusted for blood haemoglobin, *LCI* lung clearance index, *R*_*5–20*_ resistance at 5–20 Hz, *AX*^*0.5*^ square root of the area of reactance, *IQR* Inter quartile range

**Table 4:** Proportion of subjects in each group with spirometry and multiple breath wash-out measures below −1.64 z-scores

	BPD *n* = 26	Preterm *n* = 23	Asthma *n* = 23	Healthy controls *n* = 24
FEV_1_ *z-scores*	5 (19)*	0	0	0
FVC *z-scores*	0	1 (4.3)	0	0
FEV_1_/FVC *z-scores*	7 (27)**	1 (4.3)	1 (4.3)	0
LCI *z-scores*	8 (32)***	2 (8.7)	4 (17)	1 (4.6)

BPD-, preterm- and asthma-groups were compared to healthy controls. Data are presented as numbers (%). FEV_1_, FVC, FEV_1_/FVC: post bronchodilator measures. *p ≤ 0.05; **p ≤ 0.01; ***p ≤ 0.001. Abbreviations: *BPD* bronchopulmonary dysplasia, *FEV*_*1*_ forced expiratory volume in 1 s, *FVC* forced vital capacity, *LCI* lung clearance index

The BPD group had significantly lower lung volumes than the other groups (Table 3). Both TLC and VC were decreased (*p* < 0.05 and *p* < 0.01) although no significant differences were observed when comparing the ratio between the RV and TLC.

Both the BPD- and the preterm groups had decreased D_LCO_ compared to healthy controls (*p* < 0.001, Table 3, Fig. 2c). Seventy-three percent of the BPD group and 56% of the preterm group were positive in methacholine challenge test (Table 3). By study design, everyone in the asthma group and none of the healthy controls had a positive methacholine challenge test.

Impulse oscillometry showed no significant differences when comparing R_5–20_ and AX^0.5^ between the four groups (Table 3). In the BPD group LCI was significantly (*p* < 0.001) higher compared to the other groups (Table 3; Fig. 2d), but there were no statistically significant differences between preterm-, asthma groups and healthy controls. Thirty-two percent of the participants in the BPD group had LCI measures above lower limit of normal (≤ − 1.64 z-scores). The corresponding percentage in the preterm group was 8.7%, in the asthma group 17% and in healthy controls 4.6% (Table 4).

### Correlations between lung function, perinatal characteristics, HRQoL and symptoms

Within the BPD- and preterm groups we found no statistically significant associations between FEV_1,_ FVC, FEV_1_/FVC ratio, TLC, VC, RV, D_LCO_, LCI, R_5–20_, and AX to GA, BW or SGA. We could not show any statistically significant correlations between scores in SGRQ and SF-36 or symptoms and lung function measures in this study.

## Discussion

This study demonstrates that adults born preterm with a prior neonatal diagnosis of BPD display signs of obstructive and restrictive lung function impairment together with gas diffusion disturbance. The lung function impairment included involvement of small airways and was different from that of asthmatics. Despite more airway obstruction from a quantitative point-of-view, the individuals in the BPD group reported less symptoms and less impact on quality of life than the asthma group. We were also able to demonstrate that preterm born young adults without prior neonatal diagnosis of BPD were normal in their lung function despite a disturbance of gas diffusion. Finally, both preterm groups, in particular those with a history of BPD had a high incidence of bronchial hyper-reactivity.

Bronchopulmonary dysplasia is a common cause of respiratory disease in children born preterm. The condition is often interpreted as a developmental disorder with pathogenesis being linked to underdeveloped lungs, inflammation, baro- and volutrauma resulting from mechanical ventilation and oxidative stress due to oxygen treatment [27]. Northway et al. [28] characterized “old” BPD by inflammation, airway smooth muscle hypertrophy, emphysema, and parenchymal fibrosis caused by high oxygen concentration and high ventilation pressures. The “new” BPD is characterized by even more immature lung tissue affected by reparative processes, impaired alveolarization, and dysmorphic vascular growth [29]. Obstructive airway impairments have previously been demonstrated in children [15], adolescents [30] and adults [31] born preterm with BPD [32, 33]. To identify preterm born patients at risk for developing chronic lung disease in adulthood is however difficult due to the lack of specific disease markers. We aimed to study pulmonary outcomes in young adults born preterm with and without a history of BPD and compare to asthmatics and healthy controls. In order to rule out potential effects from smoking and ongoing medication, we choose to only include never-smokers and to perform the clinical investigation when the study subjects had been without any anti-inflammatory treatment for at least 3 months. No subjects were excluded due to the criterion of being without anti-inflammatory treatment. We do not believe any preterm born subjects with or without BPD declined participation in the study due to not being able to use their pharmacological treatment because the relevant information was collected at inclusion. The procedure did not totally eliminate the bias of having less severely affected subjects in the study but it made it less likely.

The asthma group was well-defined with all subjects sensitized to at least one airborne allergen. They showed an increase in FeNO and blood eosinophils as a sign of ongoing inflammation and were all positive in methacholine challenge test. The asthmatics were all sensitized to pets and some of them also to other airborne allergens such as pollen and house dust mite. As shown in a previous study [34], BPD patients were to lesser extent sensitized to airborne allergens. The preterm born subjects, in particular those adults with a history of BPD were to larger degree positive in methacholine challenge test, as previously demonstrated [33, 35].

The BPD group demonstrated elevated LCI, a sign of small airway disease and air trapping. A low gas diffusing capacity was observed in the BPD group, but also in the preterm group who had normal expiratory flow volumes, confirming earlier findings [31]. We found no pronounced differences between the groups using IOS, opposite to what has been shown in previous studies with children and adolescents [15, 30]. We speculate that this is related to the relatively older population examined in the current study, as the IOS method may be more sensitive in smaller lung volume range seen in younger individuals.

The BPD group reported less symptoms compared to the asthma group. This is possibly a result of the subjects getting used to a lung function impairment lasting since the neonatal period without larger fluctuations. The asthma group reporting more symptoms might be explained by a disease with alterations in time periods and maybe by inadequately treated asthma.

In agreement to Bozzetto et al. [36], we found that the asthma group demonstrated the lowest PCS scores in SF-36 whereas the BPD group reported similar scores as the healthy controls.There were no differences in PCS scores between the BPD group and the healthy controls. This might be an effect of adaptation (i.e. adjusting the physical activity intensity to the actual lung function to avoid symptoms) as we did not detect any differences in amount of hours of physical activity from the questionnaire. In an earlier study [1], we did not find any significant differences in ergospirometry between the BPD-groups and the preterm group without BPD even if a tendency towards less work capacity was seen with increasing severity of BPD. However, to further evaluate this matter in future studies, exercise testing may be useful.

We also showed that the preterm group displayed similar PCS scores as the asthma group although the preterm group had normal lung function measures which might reflect that many of them suffer from hyper-reactive airways. Opposite to Landry et al. and Bozzetto et al. [33, 36] we found that both the BPD- and preterm groups reported decreased MCS scores compared to healthy controls.

The strength of the current study is that the included cohort was well characterized both regarding lung function and considering subjective, patient-related outcome measures. The preterm born participants in both groups were born in the time period 1992 to 1998 when transition to modern neonatal care occurred, therefor, no clear distinction between “old” and “new” BPD cases can be made. There was also a difference in GA and BW between the preterm born groups, though representative for the whole original cohort except for GA where the subjects in the BPD group was 4 days younger in mean GA at birth. In our study we were unable to confirm any correlations between being born SGA and impaired lung function which is conflicting with previous studies [31, 37]. We suspect that this could be an effect of small sample size and/or different methods to calculate SGA.

## Conclusion

In summary, we conclude that adults with a history of BPD have obstructive and restrictive lung function impairment, including involvement of small airways. Despite more airway obstruction, the BPD group has less symptoms than those with asthma. The underlying mechanisms for this remain unknown and warrant further investigation. It is important to characterize clinical features, airway symptoms and lung function in subjects born preterm in order to better understand the link between preterm birth and development of chronic lung disease in adulthood. Our study highlights the need for objective assessment of lung health in this group.

## Additional files

Additional file 1: **Figure S1.** Schematic description of recruitment to the LUNAPRE-study. (PPTX 63 kb)

Additional file 2: Supplementary methodology. (DOCX 51 kb)

Additional file 3: **Table S1.** Symptoms and habits of study participants. (DOCX 20 kb)

## Data Availability

Anonymized data from the current study is available from the corresponding author on reasonable request.

## Abbreviations

ADD: Attention deficit disorder
ADHD: Attention deficit hyperactivity disorder
AX^0.5^: Square root of the area of reactance
BMI: Body mass index
BPD: Bronchopulmonary dysplasia
COPD: Chronic obstructive lung disease
CPAP: Continuous Positive Airway Pressure
CRP: C-reactive protein
D_LCO adj_: Diffusing capacity of the lung for Carbon monoxide adjusted for blood haemoglobin
F: Female
FeNO: Fractional exhaled nitric oxide
FEV_1_: Forced expiratory volume in 1 s
FVC: Forced vital capacity
GA: Gestational age
GINA: Global Initiative for Asthma
GLI: Global Lung Initiative
ICR: Inter quartile range
LCI: Lung clearance index
M: Male
MCS: Mental component summery
N/A: Not applicable
PCS: Physical component summary
PDA: Patent Ductus Arteriosus
ppb: Parts per billion
R_5–20_: Resistance at 5–20 Hz
ROP: Retinopathy of Prematurity
RV: Residual volume
SGA: Small for gestational age
TLC: Total lung capacity
VC: Vital capacity
