# Supplementary material for: Pulmonary outcomes in adults with a history of Bronchopulmonary Dysplasia differ from patients with asthma
Source: Respir Res. 2019 May 24;20:102. doi: 10.1186/s12931-019-1075-1 (PMC6534852; doi:10.1186/s12931-019-1075-1)
Supplement: Supplementary file 1 — Figure S1. Schematic description of recruitment to the LUNAPRE-study. (PPTX 63 kb) [file 12931_2019_1075_MOESM1_ESM.pptx]

## Slide 1
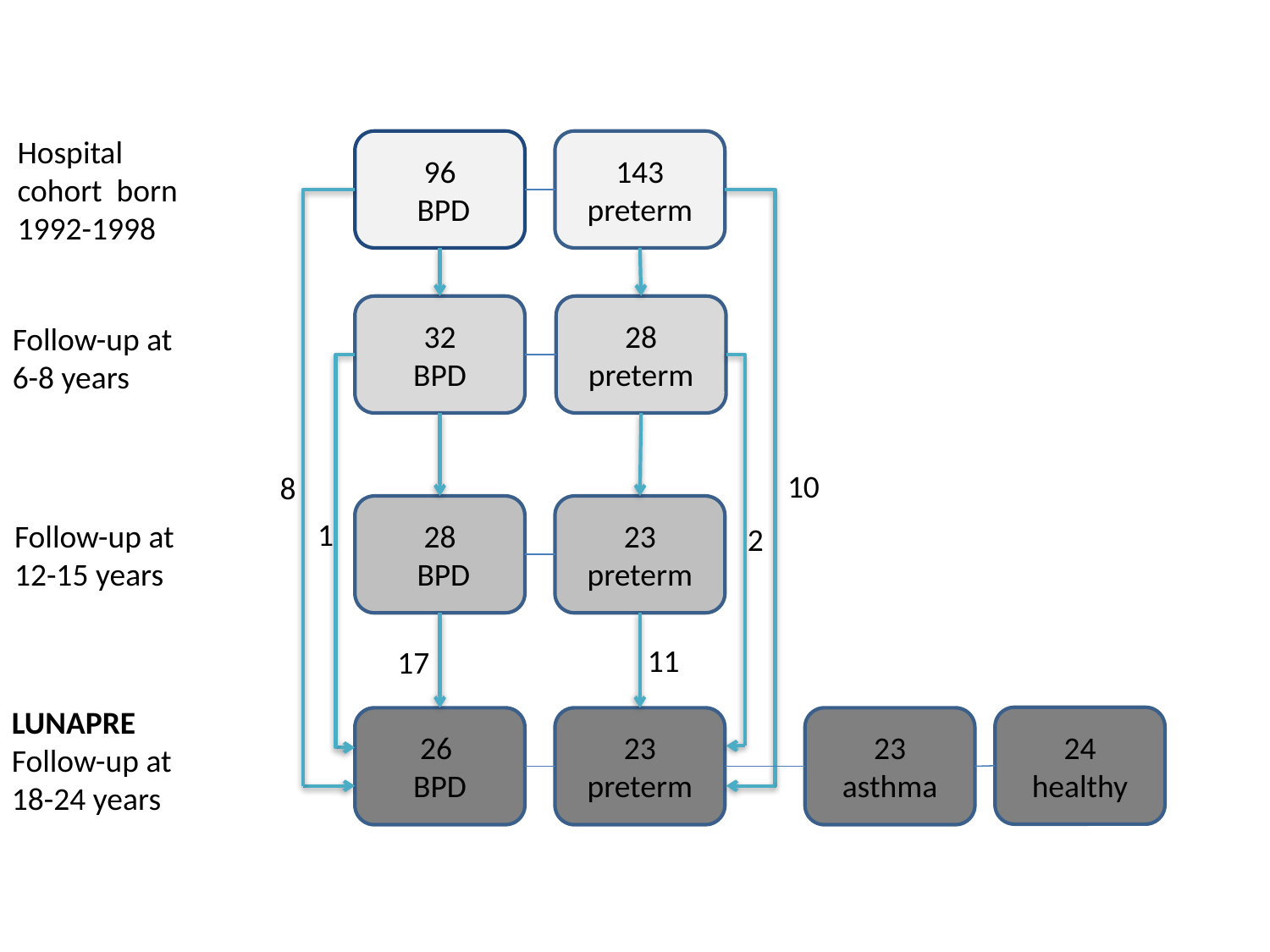

Hospital cohort born 1992-1998
96
 BPD
143 preterm
 32
BPD
28 preterm
Follow-up at
6-8 years
10
8
28
 BPD
23 preterm
1
Follow-up at
12-15 years
2
11
17
LUNAPRE
Follow-up at
18-24 years
24 healthy
26
BPD
23 preterm
23 asthma
