# Supplementary material for: Pulmonary outcomes in adults with a history of Bronchopulmonary Dysplasia differ from patients with asthma
Source: Respir Res. 2019 May 24;20:102. doi: 10.1186/s12931-019-1075-1 (PMC6534852; doi:10.1186/s12931-019-1075-1)
Supplement: Supplementary file 2 — Supplementary methodology. (DOCX 51 kb) [file 12931_2019_1075_MOESM2_ESM.docx]

**[Additional file 2:](https://static-content.springer.com/esm/art%3A10.1186%2Fs12931-018-0950-5/MediaObjects/12931_2018_950_MOESM1_ESM.docx)**

**Supplementary methodology**

**PARTICIPANTS**

We included four adult study groups: preterm born at gestational age (GA) ≤ 32 weeks with a neonatal diagnosis of BPD (BPD group) , preterm (born ≤ 32 weeks) without BPD (preterm group), patients (born ≥ 37 weeks) with asthma according to GINA guidelines[1] (asthma group) and healthy controls (born ≥ 37 weeks) as part of the LUNAPRE (**LU**Ng **A**dult **PRE**maturity) cohort (clinicaltrials.gov/ct2/show/ NCT02923648).

The preterm born participants were recruited from a pre-existing cohort [2, 3] (e-Figure 1) at the neonatal unit of Sachs’ Children and Youth Hospital, Södersjukhuset, Stockholm, Sweden, where they were admitted in the neonatal ward between 1992 and 1998. One patient in the preterm group had been admitted to the neonatal unit at Danderyd’s Hospital, Stockholm. Sachs’ Hospital serves as a regional neonatal center for the Southern part of Stockholm. The former patients were contacted and received information about the study by mail and telephone calls when telephone numbers were available. The healthy controls and patients with asthma were recruited from a student web-site in Stockholm ("Studentkaninen", http://www.studentkaninen.se) and through advertising on social media and in newspapers. All participants provided written informed consent, and the study was approved by the regional ethics committee in Stockholm (ref: 201211872-31/4).

Between 2013 and 2017, patients and controls were invited to Department of Medicine Solna, Karolinska Institutet, Department of respiratory medicine and allergy, Karolinska University Hospital Solna, Stockholm, Sweden, and at Sachs’ Children and Youth Hospital, Södersjukhuset, Stockholm.

**LUNG FUNCTION TESTING**

**Dynamic spirometry** Spirometry was measured using a Sensormedics 6200 (SensorMedics, Yorba Linda, California, USA) with the subject in the sitting position, wearing a nose-clip. All subjects performed at least three maximal expiratory flow volume (MEFV) measurements. The highest values of forced vital capacity (FVC), forced expiratory volume in 1 second (FEV_1_), and maximum expiratory flow when 50% of the FVC remains to be exhaled (FEF_50_) were extracted and used from analysis, provided that the subject’s effort was coded as being maximal by the test leader, the MEFV curve passed visual quality inspection, and that the two highest FEV_1_ and FVC readings were reproducible according to ATS/ERS criteria.[4]FEV_1_/FVC is expressed as a ratio. Reversibility to bronchodilator was tested using four doses of Airomir® (salbutamol) 0.1 mg/dose and repeating spirometry 15 minutes later. Reversibility was defined as an increase of 12% and 200 ml in FEV_1_ according to international guidelines.[4, 5]

**Body plethysmography and diffusing capacity for carbon monoxide (D_LCO_**)

Whole Body plethysmography was performed with Vmax62 J CareFusion (SensorMedics, Yorba Linda, California, USA) to measure static lung volumes and diffusing capacity. The Vmax 62 J system is fully automatized and measures dynamic lung volumes, static lung volumes, plethysmographic calculation of FRC (Functional Residual Capacity) and diffusing capacity with single-breath method. Calculations were made from three approved SVC (slow vital capacity) breaths with a variability <150 ml and ERV (expiratory reserve volume) <150 ml. Diffusing capacity measurements were made from at least two approved figures with reproducibility <10% of the mean volume or <1.0 mmol kpa/min. Diffusing capacity was correlated with haemoglobin value (Hb) using Haemocue Hb201 DM-analyser. All calculations from the body plethysmograph were made according to the international guidelines from ATS/ERS.

**Methacholine challenge test**

Bronchial hyper-responsiveness to methacholine was dosed utilizing a Spira nebulizer (Spira Elektro 2, Respiratory Care Centre, Hämeenlinna, Finland) according to modified protocol.[6, 7] Pre-challenge FEV_1_ was measured before and after inhalation of normal saline concentration with Medicro Spirometer (Ailos Medical AB, Karlstad, Sweden). If pre methacholine challenge FEV_1_ was <70% or when FEV_1_ fell more than 10% post saline inhalation the healthy control subject was excluded from the study. The aerosol delivered was adjusted to 0.5 l/s seconds, with a start volume of 100 ml and a tidal volume of 0.5-1 liter. Each subject practiced the nebulizer before the study. The mouth piece was held firmly between the teeth and a nose clip applied. Methacholine solution with concentration starting from 1 mg/ml was inhaled. Starting dose of 18μg of saline and methacholine mixture was delivered with 2 breaths with increasing dose and number of breaths every three minutes to a cumulative dose of 3520μg according to separate protocol. Spirometry for calculation of FEV_1_ was performed 2.5 min after inhalation of each dose of methacholine given. The methacholine provocation was terminated if the FEV_1_ decreased with ≥ 20% or the maximum dose of 3520μg was given.

**Impulse Oscillometry**  Impulse Oscillometry (IOS) was performed using the Jaeger MasterScreen-IOS system (Carefusion Technologies, San Diego, California). Briefly pressure impulses were sent from a loudspeaker through the respiratory system. The subjects were encouraged to breathe tidal breathing with the lips tightly sealed around the mouthpiece and supporting cheeks with their hands to avoid impulse pressure loss due to upper airway shunt. A minimum of two recordings without artefacts for at least 20 s were saved for later analysis. A coherence value >0.80 at 10 Hz was used as criteria for an appropriately performed test. The mean value of the resistance at 5 Hertz (R_5_), frequency dependence of resistance (R_5-20_) and the square root of the area of reactance (AX^0.5^) were used for analysis.[8, 9]

**Multiple breath washout**

Nitrogen dioxide multiple breath wash-out (N_2_ MBW) was performed using the Exhalyzer®D N_2_ MBWdevice (Eco Medics AG, Duernten, Switzerland). Two technically acceptable N_2_ MBW tests were performed in all subjects, in accordance with the recently published studies.[10, 11] Mean values for LCI were extracted.

**Fractional exhaled Nitric Oxide**

Fractional exhaled Nitric Oxide *(*FeNO) was measured with a chemiluminescence analyzer (EcoMedics Exhalyzer® CLD 88sp with Denox 88, Eco Medics, Duernten, Switzerland). The procedure was performed in accordance with published guidelines.[12] Mean exhalation flow rate was 50 mL/s ± 10% during the NO plateau. The maneuver was repeated until two exhalations agreed to within 5% coefficient of variation (CV), or three exhalations agreed to within 10% CV. The NO concentration, FeNO, was defined as the mean of these values expressed in parts per billion (ppb). The analyzer was calibrated using a standard NO calibration gas (Air Liquide Deutschland GmbH, Krefeld, Germany). In a few study subjects FeNO was measured using a NIOX device (Aerocrine AB, Solna, Sweden). [13]

**REFERENCES**

1.GINA. Global strategy for asthma management and prevention (updated 2017): Global Initiative for Asthma (GINA).<http://www.ginasthma.org>. 2017.

2.Brostrom EB, Thunqvist P, Adenfelt G, Borling E, Katz-Salamon M. Obstructive lung disease in children with mild to severe BPD. *Respiratory Medicine* 2010: 104(3): 362-370.

3.Um-Bergstrom P, Hallberg J, Thunqvist P, Berggren-Brostrom E, Anderson M, Adenfelt G, Lilja G, Ferrara G, Skold CM, Melen E. Lung function development after preterm birth in relation to severity of Bronchopulmonary dysplasia. *BMC pulmonary medicine* 2017: 17(1): 97.

4.Miller MR, Hankinson J, Brusasco V, Burgos F, Casaburi R, Coates A, Crapo R, Enright P, van der Grinten CPM, Gustafsson P, Jensen R, Johnson DC, MacIntyre N, McKay R, Navajas D, Pedersen OF, Pellegrino R, Viegi G, Wanger J. Standardisation of spirometry. *European Respiratory Journal* 2005: 26(2): 319-338.

5.Pellegrino R, Viegi G, Brusasco V, Crapo RO, Burgos F, Casaburi R, Coates A, van der Grinten CP, Gustafsson P, Hankinson J, Jensen R, Johnson DC, MacIntyre N, McKay R, Miller MR, Navajas D, Pedersen OF, Wanger J. Interpretative strategies for lung function tests. *The European respiratory journal* 2005: 26(5): 948-968.

6.Nieminen MM, Lahdensuo A, Kellomaeki L, Karvonen J, Muittari A. Methacholine bronchial challenge using a dosimeter with controlled tidal breathing. *Thorax* 1988: 43(11): 896-900.

7.O'Connor G, Sparrow D, Taylor D, Segal M, Weiss S. Analysis of dose-response curves to methacholine. An approach suitable for population studies. *The American review of respiratory disease* 1987: 136(6): 1412-1417.

8.Oostveen E, MacLeod D, Lorino H, Farre R, Hantos Z, Desager K, Marchal F. The forced oscillation technique in clinical practice: methodology, recommendations and future developments. *The European respiratory journal* 2003: 22(6): 1026-1041.

9.Al-Mutairi SS, Sharma PN, Al-Alawi A, Al-Deen JS. Impulse oscillometry: an alternative modality to the conventional pulmonary function test to categorise obstructive pulmonary disorders. *Clinical and experimental medicine* 2007: 7(2): 56-64.

10.Robinson PD, Latzin P, Verbanck S, Hall GL, Horsley A, Gappa M, Thamrin C, Arets HG, Aurora P, Fuchs SI, King GG, Lum S, Macleod K, Paiva M, Pillow JJ, Ranganathan S, Ratjen F, Singer F, Sonnappa S, Stocks J, Subbarao P, Thompson BR, Gustafsson PM. Consensus statement for inert gas washout measurement using multiple- and single- breath tests. *The European respiratory journal* 2013: 41(3): 507-522.

11.Kjellberg S, Houltz BK, Zetterstrom O, Robinson PD, Gustafsson PM. Clinical characteristics of adult asthma associated with small airway dysfunction. *Respir Med* 2016: 117: 92-102.

12.Dweik RA, Boggs PB, Erzurum SC, Irvin CG, Leigh MW, Lundberg JO, Olin AC, Plummer AL, Taylor DR. An official ATS clinical practice guideline: interpretation of exhaled nitric oxide levels (FENO) for clinical applications. *Am J Respir Crit Care Med* 2011: 184(5): 602-615.

13.ATS/ERS recommendations for standardized procedures for the online and offline measurement of exhaled lower respiratory nitric oxide and nasal nitric oxide, 2005. *Am J Respir Crit Care Med* 2005: 171(8): 912-930.
