# Supplementary material for: Pulmonary outcomes in adults with a history of Bronchopulmonary Dysplasia differ from patients with asthma
Source: Respir Res. 2019 May 24;20:102. doi: 10.1186/s12931-019-1075-1 (PMC6534852; doi:10.1186/s12931-019-1075-1)
Supplement: Supplementary file 3 — Table S1. Symptoms and habits of study participants. (DOCX 20 kb) [file 12931_2019_1075_MOESM3_ESM.docx]

**[Additional file 3:](https://static-content.springer.com/esm/art%3A10.1186%2Fs12931-018-0950-5/MediaObjects/12931_2018_950_MOESM1_ESM.docx)**

**Supplementary Table 1. Symptoms and habits of study participants**

|  | BPD  *n*=26 | Preterm  *n*=23 | Asthma  *n*=23 | Healthy controls  *n*=24 |
| --- | --- | --- | --- | --- |
| Screen-time (TV, computer) >5 hours/day | 8(31) | 5(22) | 2(9)* | 11(46) |
| Physical activities  >3 hours/week | 11(42) | 11(48) | 15(65) | 12(50) |
| Sleeping hours | 7 (6; 10) | 7 (4; 8) | 7 (5; 9) | 7 (6; 11) |
| Having episodes of wheeze | 5(19) | 1(4) | 15(65)*** | 2(8) |
| Experiencing episodes of cough | 9(35) | 2(9) | 14(61)*** | 3(13) |
| Experiencing episodes of breathlessness | 11(42)** | 6(26)* | 21(91)*** | 1(4) |
| Having phlegm hard to expectorate | 4(36)* | 3(13) | 4(17)* | 0 |
| Ever had pneumonia | 6(23) | 2(9) | 2(9) | 5(21) |
| Any antibiotics the past 12 months | 0 | 3(13) | 4(17)* | 0 |
| SF-36 PCS | 56.3  (53.2; 57.6) | 52.8***  (49.4; 55.1) | 53.5**  (49.1; 57.0) | 57.6  (55.7, 58.4) |
| SF-36 MCS | 48.2*  (40.4; 50.8) | 44.2*  (40.6; 52.5) | 48.3  (40.6; 53.2) | 51.7  (48.3; 53.9) |
| SF-36 PF | 100  (90; 100) | 100  (90; 100) | 95***  (87.5; 95) | 100  (97.5; 100) |
| SF-36 RP | 100  (100-100) | 100*  (75-100) | 100*  (87.5-100) | 100  (100-100) |
| SF-36 BP | 84  (74; 100) | 74***  (62; 84) | 100  (68; 100) | 100  (84; 100) |
| SF-36 GH | 77***  (67; 85) | 71***  (57; 85) | 74.5**  (47; 92) | 92  (86; 100) |
| SF-36 VT | 65**  (50; 75) | 50**  (45; 65) | 65*  (55; 72.5) | 75  (70; 80) |
| SF-36 SF | 93.8*  (75; 100) | 100*  (75; 100) | 100  (87.5; 100) | 100  (100; 100) |
| SF-36 RE | 100**  (66,7; 100) | 100**  (66,7; 100) | 100*  (66,7; 100) | 100  (100; 100) |
| SF-36 MH | 76  (68; 84) | 76  (64; 88) | 76  (60; 86) | 80  (72; 92) |
| SGRQ Total score | 6.3  (0; 39.2) | 4.9  (1.2; 14.6) | 16.5***  (2.8; 41.4) | 3.8  (0.8; 11.3) |
| SGRQ Symptom score | 11.4  (0; 20) | 8.6*  (0; 23) | 27.8***  (19.5; 42.5) | 5.5  (0; 8.6) |
| SGRQ Activity score | 12.2  (6; 18.2) | 9.1  (6; 12.2) | 20.9***  (12.2; 29.3) | 6  (6; 12.2) |
| SGRQ Impact score | 0*  (0; 4.8) | 0  (0; 0) | 11.2***  (2; 16.8) | 0  (0; 0) |

Data are presented as median (IQR) or numbers (%).*: p ≤0.05; **: p ≤ 0.01; ***: p ≤0.001, comparing BPD-, preterm- and asthma groups to healthy controls. Abbreviations: BPD: bronchopulmonary dysplasia; IQR: inter quartile range SF-36: short form health survey; PCS: physical component summery; MCS: mental component summery; PF: physical functioning; RP: role physical; BP: bodily pain; GH: general health; VT: vitality; SF: social functioning; RE: role emotional; MH: mental health; SGRQ: St George’s respiratory questionnaire
